# Parallel biocomputing

**DOI:** 10.1186/1751-0473-6-4

**Published:** 2011-03-18

**Authors:** Kenneth S Kompass, Thomas J Hoffmann, John S Witte

**Affiliations:** 1 Department of Epidemiology and Biostatistics and Institute for Human Genetics, University of California San Francisco, San Francisco, CA, USA

## Abstract

**Background:**

With the advent of high throughput genomics and high-resolution imaging techniques, there is a growing necessity in biology and medicine for parallel computing, and with the low cost of computing, it is now cost-effective for even small labs or individuals to build their own personal computation cluster.

**Methods:**

Here we briefly describe how to use commodity hardware to build a low-cost, high-performance compute cluster, and provide an in-depth example and sample code for parallel execution of R jobs using MOSIX, a mature extension of the Linux kernel for parallel computing. A similar process can be used with other cluster platform software.

**Results:**

As a statistical genetics example, we use our cluster to run a simulated eQTL experiment. Because eQTL is computationally intensive, and is conceptually easy to parallelize, like many statistics/genetics applications, parallel execution with MOSIX gives a linear speedup in analysis time with little additional effort.

**Conclusions:**

We have used MOSIX to run a wide variety of software programs in parallel with good results. The limitations and benefits of using MOSIX are discussed and compared to other platforms.

## Background

With the widespread use of high-throughput genomic technologies, there is currently a great interest in running these computations in parallel. The last few years have seen a sharp increase in the number of both freely and commercially available packages for parallel computing. For a good overview of parallel computing in R see [1] and the R task page for parallel computing [2]. These packages generally run across a single CPU with multiple cores [3], multiple CPUs or cores (e.g., snowfall and sfCluster [4]), and there is also progress on using graphics processors for parallelization with gputools [5]. Some of these try to automate the parallelization process, but generally still require some setup, and require no modification to shared variables. They also require you to have cluster software installed, such as LSF [6], or Rocks [7], to name a few. Here we report good success on building and running a cluster with a more universal and mature tool, MOSIX [8]. MOSIX is a linux kernel patch that, once compiled and installed on a cluster, provides single system image (SSI) parallel computation.

The steps required by the user to use MOSIX are conceptually the same as for parallel computing on a wide variety of platforms, e.g., MPI-based [9]. That is, within a session, data objects are created, saved to disk, and sent out to other nodes for parallel processing. Results are transmitted back to, and aggregated at, the originating node. Generally, no modifications to existing code are necessary other than to the calling script.

## Methods

We assembled a small compute cluster and tailored it for computing a wide variety of statistical genetics problems. Our compute cluster contains 14 commodity quad-core computers (for a total of 56 simultaneous parallel jobs) with varying amounts of memory (two boxes have very large amounts of memory to handle some problems), and a fast hard drive. However, one can tailor the specs of each node to one's own needs, as long as the computer supports the modern Linux kernel, which most will. Communication between nodes is through the fastest commodity type ethernet switch/router available (we used a router to have one fixed IP address with port forwarding to each of the nodes). Installation instructions for MOSIX are available online [10]. Briefly, we configured the first machine by installing a Linux operating system, setting up a fixed local IP address, installing a kernel patch, setting a passphrase, and opening up a range of IP addresses through MOSIX. We then used the open source drive imaging software [11] to clone this image to each of the other nodes, and then changed the fixed IP address for each node. Our installation of MOSIX on 64-bit openSUSE linux v11.1 was simple with the MOSIX binary kernel rpm installer. Other linux distributions took more effort and were not as successful as openSUSE when building the patched kernel.

Rather than keeping users' files on the compute nodes' disks, we had a separate network disk storage array. Transfers are typically done via scp, sftp, sshd, or rsync. We found sshd to be the simplest and most convenient for accessing the networked disk from the compute nodes, although better performance can be achieved through setting up a network file system. Alternatively, one can simply have one node with a larger storage capacity, and run jobs from that node and/or use sshd to mount it on all other nodes.

## Results and Discussion

In the previous section we described how to build a cluster from commodity hardware. Next, we show how to run a statistical genetics problem in parallel across a cluster with MOSIX to reduce the computation time by the total number of cores in a cluster for a computationally intensive statistical genetics problem.

### A genetics case study example

Higher resolution and lower cost genotyping arrays have ushered in large studies in humans called Genome Wide Association Studies, or GWAS [12]. A popular GWAS goal is the identification of single nucleotide polymorphisms, or SNPs, which are variable bases in DNA sequences, that might predispose or alter the progression of disease. This is done by statistically associating disease phenotypes with SNPs. A GWAS can also link many other phenotypes to SNPs. One commonly studied, continuous phenotype is the messenger RNA expression level of a gene. An eQTL (for expression quantitative trait locus) study can identify SNPs that affect the messenger RNA levels (so-called expression traits) of protein-coding genes [13]. eQTL may propose a mechanism when SNPs associated with disease have no known function, which is often the case with SNPs in noncoding DNA regions. Such SNPs have been shown to affect expression levels of genes with key pathological roles [14]. Thus, an adequately-powered eQTL study could determine whether a SNP associated with disease also affected the expression levels of any known protein coding genes, and suggest a mechanistic link for further wet-laboratory experimentation. Because an eQTL experiment generally involves thousands of expression traits as the phenotypes of interest, it is generally more computationally intensive than single-phenotype designs.

Here we provide an example eQTL analysis with simulated data using code in the popular open source language R [15]. This is a scalable method for parallelizing many tasks on a MOSIX-enabled computing network. Together, this example has approximately 25 lines of extra R code to parallelize the eQTL testing on a MOSIX-enabled system, and can be easily adapted to run other tasks or be coded in other languages. Other utilities, such as 'batch' [16], can be used to streamline parallel execution via MOSIX. In our example, we first simulate a dataset with 100 samples, 1000 genotyped loci, and 1000 genes for eQTL association testing.

g.calls = data.frame(matrix(sample(c(rep(0,500), rep(1,500), rep(2,500)), 100*1000,

   *replace = TRUE), nrow = 1000)) ## row: genotype calls, column: samples*

exprs <- matrix(rnorm(100*1000, 0, 1), nrow = 1000) # row: expression, column: sample

In this code, we first simulate a matrix of single nucleotide polymorphism (SNP) calls in g.calls with an additive coding, i.e., a count of the number of minor alleles an individual has at a locus. We then simulate a matrix of random mRNA expression data (a quantitative trait).

Next, we evenly split the job into batches to be run across the nodes via the function chopper. This helper function creates a string of the beginning and ending indices to the SNPs, starting at beg.index (1 in our example), ending at end.index (the number of genotyped loci in our example), for batches jobs (the number of cores in the cluster).

*chopper = function(beg.index, end.index, batches){*

   *a = seq(beg.index, end.index, round((end.index-beg.index)/batches))*

   *b = unique(append(a[2:length(a)]-1, end.index))*

*return(paste(a, b, sep = "_"))*

}

The chopper function is important because each node should process only a small portion of the genotype calls from g.calls. In real examples, one may also wish to chop the dataset into pieces as well. Next, we create a function that will process each of these datasets on each remote node.

*par.fx = function(o.file) {*

   *o.f = file(paste(o.file, "_p_val.temp", sep = ""), 'w') # output file*

   *gcr = sort(as.integer(unlist(strsplit(o.file, '_')))) # start/stop*

   *apply(g.calls[(gcr[1]:gcr[2]),], 1*,

      *function(x){*

         *p.vector = apply(exprs, 1, function(y){summary(lm(y ^~^as.integer(x)))$coeff[2, 4]})*

         *cat(as.character(p.vector), file = o.f, sep = '\n')*

      })

}

The function par.fx accepts one parameter telling it what portion (i.e., which rows) of g.calls to process, creates an intermediate p-value file, does the actual association testing (an ANOVA of gene expression on genotype), and prints p-values to the intermediate file. Then we write out the dataset g.calls and the function to analyze the dataset par.fx out to disk.

*save(g.calls, exprs, par.fx, file="Image.RData")*

The amount of output typically generated by our computations (e.g., p-values) usually cannot be kept in memory and must be written to a file connection during computation.

Our next piece of code creates the start/stop indices for analysis, and then creates R script files that will be subsequently batched off. These scripts load in the "Image.RData" file created in the code above, and process part of it.

*batch.subsets <- chopper(beg.index = 1, end.index = nrow(g.calls), batches = 56)*

*r.scripts = sapply(batch.subsets*,

   *function(x) {*

      *batch.script = file(paste("batch", "_", x, ".R", sep = ""), "w")*

      *cat("load(", "\'", "Image.RData", "\' ", ")\n", sep = "", file = batch.script)*

      *cat("par.fx(o.file=", "\'", x, "\'", ")\n", sep = "", file = batch.script)*

      *return(summary(batch.script)$description)*

      *close(batch.script)*

   })

Once these R script files are created, we then create a shell script for the MOSIX jobs, and run them with the command system.

*shell.script <- file(paste(getwd(), "/", "Main.sh", sep = ""), "w")*

*for(i in 1:(length(r.scripts)))*

   *cat("mosrun -e -b -q20 R --vanilla < ", r.scripts[i], " &\n", sep = "", file = shell.script) quiet = system(paste("source ", summary(shell.script)$description, sep = ""), intern = TRUE*)

Lastly, once all of these scripts have finished running, we read in the intermediate files used, combine them into one, and clean up with the following code.

*p.file = file("p.values.txt", "w")*

*t.p.files = list.files(getwd(), pattern="_p_val.temp") # files with ANOVA p-values*

*t.r.files = list.files(getwd(), pattern="batch_") # intermediate scripts for(i in 1:length(t.p.files)) {*

   *cat(readLines(t.p.files[i]), file = p.file, sep = "\n") # input p-values, output to p.file file.remove(t.p.files[i], t.r.files[i]) # delete intermediate files*

*}*

*file.remove("Image.RData", "Main.sh")*

While there is relatively little penalty for I/O of parallel jobs with MOSIX across a gigabit network switch when compared to running locally, we found it wise to estimate the I/O required for each process when the per-core files to be transferred exceeded 20 MB, as MOSIX does not monitor I/O when batching jobs. This can be easily accomplished by doing a trial run with one batch and observing the transfer time for the image to be loaded into memory of the executing node. Once a delay time is known, adding the sleep = N (where N is the number of seconds to delay before executing the next line) shell command to the main shell script before each mosrun call will force a delay between the start of MOSIX jobs. We have found this to be adequate in preventing I/O overloads for loading a large R image. Because adding a simple short delay to our script prevented any I/O overload, there was no need to resort to higher-throughput technologies for I/O between nodes. Although MOSIX jobs migrate away to be executed on free nodes, output files exist only on the originating node, making collection of results very easy.

## Discussion

In the example, we used commodity hardware with MOSIX to efficiently adapt our existing R code for statistical genetics and run it in parallel. How to parallelize depends on the exact task. Generally, it is best to divide up the jobs such that each node actually processes multiple batches; in the example, each node was assigned only one job, but g.calls could have been divided up into more groups than 56, with fewer genotypes processed per batch. This is mandatory when batches may take varied or unknown periods of time for completion, as once a job has been sent to a node, it remains there unless it crashes, finishes, or a faster node becomes available.

Analyzing eQTL is computationally intensive due to the high density of the genotyping arrays, which are capable of genotyping, or calling, hundreds of thousands or even millions of SNPs, and large number of phenotypes for association with each SNP. Gene expression microarrays can detect mRNA levels of most known genes, with newer technologies able to discriminate between exons or splice variants, and a typical expression microarray might successfully measure 30,000 expression traits in *Homo sapiens*. eQTL associations can be identified by ANOVA, where the expression level of a gene is compared across genotypes at each individual genotyped locus. Thus, if 500,000 SNPs have been genotyped, and 30,000 expression traits are tested at each SNP, 15 billion ANOVAs must be computed. Given even a small cohort of 100 individuals, this would take over one year on a single, high-end PC processor. By running the tests in parallel, the time to complete this analysis can be reduced linearly to hours. Using multi-core processors aids efficiency; both the linux kernel and MOSIX can utilize each core, greatly reducing equipment footprint, cost, and power consumption. MOSIX can also be used within a single computer with a multi-core processor(s) to distribute jobs across the cores. In this case, as before, all cores have access to the available system memory within the computer.

Several aspects of MOSIX make it well-suited for parallel biocomputing; perhaps most attractively, it is stable, free for academic use, and easy to set up. Other strengths are its maturity, active development, and process migration (e.g., crashed jobs are automatically migrated to another node with no intervention required by the user). Depending on configuration, e.g., if programs will not be run natively, they need only be installed on a single node to run on the entire cluster, simplifying administration. In our experience, MOSIX was quite easy to install, had little penalty for moderate disk I/O between nodes, and was generally transparent to R and other commonly used genetics programs, such as plink [17]. For heavy I/O jobs, each node can still be used directly. Because it requires no special hardware and is compatible with low-cost, multi-core PCs, it is possible to start with a small cluster and expand as needs grow. As for any cluster, it is wise to be aware of power and cooling requirements before expanding.

If extra hardware is not available, a group of user workstations can also be linked together, which may be useful if those machines have processors which are otherwise underutilized. It is possible to have different processor architectures within the same MOSIX cluster, within certain limits. Even if most users within a group are not comfortable running linux, the more common commercial operating systems can generally be easily installed on openSUSE by the use of virtualization software like Xen [18], VirtualBox [19], or VMware [20], for example. This allows the user transparent access to their operating system of choice while allowing MOSIX access to their excess processing power. The security, free cost, and increased scientific utility of a modern POSIX operating system are side benefits.

Our cluster frees members of our lab from having to maintain their own computers and software installations. We have one faster box within the cluster, allowing us to exploit a very handy feature of MOSIX, especially when running a smaller number of jobs; interactive processes (as well as non-interactive ones) will run automatically on the fastest node regardless of the originating node where the user is logged in. This is done by adding the -b parameter when starting a program. In the case of R, typing

*mosrun -eb R --vanilla*

from the shell will start an interactive R process on the fastest available node.

There are some drawbacks to MOSIX, and many are certainly strengths of other platforms, so MOSIX will not fit everyone's needs. For example, MOSIX currently has only a basic job scheduler that queues jobs with different priorities. Higher priority jobs cannot pre-empt currently running lower priority jobs, and there is no ability to enforce user-specific limits, as in, e.g., LSF [6]. We also experimented with overclocking the processors in our cluster, but found that the MOSIX scheduler was unable to correctly batch our jobs when even one node was overclocked. MOSIX is commercial software; although at the time of writing, it is free for academic use. With MOSIX, memory cannot be shared across different jobs; each job has access to as much memory as can be allocated on the machine it is running on, and must allow for enough free memory for other jobs on that machine to run. Careful batching of data into reasonable chunks and writing efficient R code are still required (e.g., it would be inefficient to assemble a cluster and utilize its resources interpreting R code contained within large for loops). While MOSIX cannot benefit from the marginal performance gain of hyperthreading-enabled processors, it does utilize multiple cores of modern CPUs, allowing a linear increase in computing power with the number of cores.

## Conclusions

We have described the implementation of a very efficient and scalable system for parallel execution of scientific computing jobs. At the center of this is the MOSIX kernel patch, which we have used successfully for queuing various jobs with minimal overhead and nearly zero downtime. Because of the growing need for parallel computing in diverse areas of biology and medicine, many others would likely benefit from implementing similar systems running MOSIX.

## Competing interests

The authors declare that they have no competing interests.

## Authors' contributions

KSK and TJH built the cluster. KSK and TJH wrote the manuscript with input from JSW. All authors read and approved the final manuscript.
